# Avirulent Marek’s Disease Virus Type 1 Strain 814 Vectored Vaccine Expressing Avian Influenza (AI) Virus H5 Haemagglutinin Induced Better Protection Than Turkey Herpesvirus Vectored AI Vaccine

**DOI:** 10.1371/journal.pone.0053340

**Published:** 2013-01-03

**Authors:** Hongyu Cui, Hongbo Gao, Xianlan Cui, Yan Zhao, Xingming Shi, Qiaoling Li, Shuai Yan, Ming Gao, Mei Wang, Changjun Liu, Yunfeng Wang

**Affiliations:** 1 Division of Avian Infectious Diseases, State Key Laboratory of Veterinary Biotechnology, Harbin Veterinary Research Institute, The Chinese Academy of Agricultural Sciences, Harbin, China; 2 Animal Health Laboratory, Department of Primary Industries, Parks, Water and Environment, Tasmania, Australia; Federal University of Pelotas, Brazil

## Abstract

**Background:**

Herpesvirus of turkey (HVT) as a vector to express the haemagglutinin (HA) of avian influenza virus (AIV) H5 was developed and its protection against lethal Marek’s disease virus (MDV) and highly pathogenic AIV (HPAIV) challenges was evaluated previously. It is well-known that avirulemt MDV type 1 vaccines are more effective than HVT in prevention of lethal MDV infection. To further increase protective efficacy against HPAIV and lethal MDV, a recombinant MDV type 1 strain 814 was developed to express HA gene of HPAIV H5N1.

**Methodology/Principal Findings:**

A recombinant MDV-1 strain 814 expressing HA gene of HPAIV H5N1 virus A/goose/Guangdong/3/96 at the US2 site (rMDV-HA) was developed under the control of a human CMV immediate-early promoter. The HA expression in the rMDV-HA was tested by immunofluorescence and Western blot analyses, and *in vitro* and *in vivo* growth properties of rMDV-HA were also analyzed. Furthermore, we evaluated and compared the protective immunity of rMDV-HA and previously constructed rHVT-HA against HPAIV and lethal MDV. Vaccination of chickens with rMDV-HA induced 80% protection against HPAIV, which was better than the protection rate by rHVT-HA (66.7%). In the animal study with MDV challenge, chickens immunized with rMDV-HA were completely protected against virulent MDV strain J-1 whereas rHVT-HA only induced 80% protection with the same challenge dose.

**Conclusions/Significance:**

The rMDV-HA vaccine was more effective than rHVT-HA vaccine for protection against lethal MDV and HPAIV challenges. Therefore, avirulent MDV type 1 vaccine is a better vector than HVT for development of a recombinant live virus vaccine against virulent MDV and HPAIV in poultry.

## Introduction

Avian influenza (AI) is a highly contagious, re-emerging infectious disease affecting poultry worldwide, which is caused by highly pathogenic avian influenza virus (HPAIV). Avian influenza virus (AIV) encodes 11 viral proteins [1]. The most immunogenic and also most variable gene products of AIV are the envelope glycoprotein haemagglutinin (HA, 16 subtypes) and neuraminidase (NA, 9 subtypes) [2]. HPAIVs are restricted to AIV subtypes H5 and H7 and lead to generalized infections resulting in mortality as high as 100% in chickens and other susceptible domestic poultry species, although not all H5 and H7 viruses cause HPAI [3]. Except for being endemic in poultry, some AIV H5N1 viruses were also reported possessing a considerable zoonotic potential, since they already caused human infections, even to death, in 15 countries [4]. Under these circumstances, vaccination against AIV provides invaluable support to increase the host resistance and reduce environmental contamination [5]. It is believed that inactivated whole AIV virus vaccines effectively prevent AIV infection, but they also induce immune responses to nucleoprotein (NP) antigen of AIV, which interferes with epidemiological surveillance by prohibiting direct serological distinction between vaccinated and field-exposed chickens [6]. To overcome this disadvantage and facilitate differentiation of vaccinated chickens from infected chickens, DNA vaccine and virally vectored recombinant vaccines have been developed, which express one (HA) or two (HA and NA) immunogenic AIV proteins [7], [8], [9], [10], [11], [12].

The virulent Marek’s disease virus serotype 1 (MDV-1) is the etiological agent of MD and classified in the genus *Mardivirus* of the subfamily *Alphaherpesvirinae* along with two other non-oncogenic poultry viruses, Gallid herpesvirus 3 (MDV serotype 2) and serotype 3 herpesvirus of turkey (HVT, Meleagrid herpesvirus 1). Virulent MDV-1 results in a highly contagious neoplastic disease in chickens. The other two nonpathogenic members are antigenetically related to MDV-1 [13]. With introduction of HVT vaccines in the early 1970s, MD was prevented and controlled effectively. However, with increasing virulence of pathogenic MDV strains, HVT vaccine could not induce full protection against the lethal MDV any more in some regions. Nonpathogenic strains of MDV-1 like CVI988/Rispens have been proven to provide the best protection against MD due to its close genetic relatedness to MDV-1 oncogenic strains [14]. Like cell-associated MDV-1 vaccine strain CVI 988, attenuated MDV-1 strain 814 is also cell-associated, which is widely used in China as a very important vaccine for prevention of current MDV infection in Mainland China [15], [16], [17].

In the past twenty years, many virally-vectored antigen delivery systems have been developed for making recombinant vaccines for poultry. Infectious laryngotracheitis virus (ILTV) [2], HVT [18], Marek’s disease virus type 1 (MDV-1) [19], Newcastle disease virus (NDV) [20], and fowl pox virus (FPV) [21] have attracted considerable attention as antigen delivery systems since these viruses have restricted host ranges for avian species. As the first successful live vaccine to control MD, herpesvirus of turkey (HVT) has been used for a long time for protection against MD and HVT-vectored antigen delivery systems have been developed for making recombinant viral vaccines since Morgan *et al*. (1992) developed a recombinant HVT vaccine expressing the NDV fusion protein [22]. HVT vectored vaccine against both MD and IBD expressing VP2 of infectious bursal disease virus (IBDV) was shown to be effective and safe, which can be inoculated into embryonated eggs and 1-day chickens and is effective in the presence of high titers of maternally derived antibodies [23], [24], [25]. The resulting vaccine Vaxxitex^R^HVT+IBD was licensed as a commercialized animal herpesvirus vector vaccine product. rHVT vaccines expressing NDV haemagglutinin neuraminidase (HN) and the fusion protein (F) were also developed against ND [26], [27].

With emergence of strains of increased virulence of MDV-1, HVT no longer provides good protection against the virulent MDVs. Avirulent MDV serotype 1 strains were introduced as MD vaccines to protect chickens more effectively for control of MD and MDV1 vaccine strains have been used to develop recombinant MDV vaccines. Sakaguchi *et al*. generated the first recombinant MDV vaccine expressing the F gene of NDV, which induced sufficient protection against NDV and MDV challenges in the commercial chickens with maternal antibodies [28]. Recombinant MDV vaccine expressing IBDV VP2 gene induced effective protection against very virulent IBDV (vvIBDV) challenge and full protection against very virulent MDV (vvMDV). MDV-1 strain CVI988 was used as a vector to express IBDV VP2 gene at different gene locus, respectively [19]. Complete protection against virulent MDV and solid protection against virulent IBDV were induced in the immunized chickens, revealing the potential of MDV-1 vectored vaccines [19], [29]. These studies have revealed the more effective protection of recombinant MDV-1 vaccines expressing the protective genes of other viruses than HVT vectored vaccines.

The HVT vectored AI vaccines were generated and their protective efficacy evaluated recently. Recombinant HVT expressing AIV H7HA provided efficient protection against HPAIV H7 virus and virulent MDV [18]. Our previous study indicated that recombinant HVT (rHVT) expressing AIV H5HA at US2 gene insertion site is more effective for foreign gene expression compared to expression at US 10 gene insertion site [30]. To date there are few data on the protective efficacy of recombinant MDV-1 expressing AIV antigens against HPAIV even though recombinant MDV-1 vaccines may be more effective than HVT. To confirm whether attenuated MDV-1 strains are more effective than HVT vaccine strain as vectors for development of recombinant live virus vaccines against MDV and AIV in poultry, we constructed an rMDV-HA expressing HA gene of AIV H5HA and its immune efficacy against HPAIV and virulent MDV evaluated. An H5 subtype HA gene was inserted at the US2 gene locus of attenuated MDV vaccine strain 814 for development of rMDV-HA and the results of the chicken vaccine efficacy trial indicated that rMDV-HA conferred more effective protection against HPAIV and MDV challenges than rHVT-HA.

## Results

### Generation, Purification and Verification of rMDV-HA

**Figure 1:**
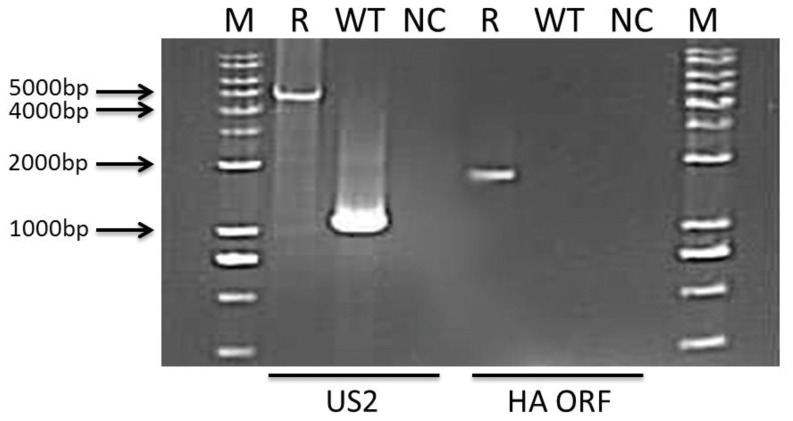
Validation of rMDV-HA by PCR amplifications of MDV US2 gene region and HA open reading frame. (M) DNA ladder; (R) the rMDV-HA infected CEF cells: the fragment of US2 region that covered the HA expression cassette and gpt selective marker, 4572 bp; the fragment of HA open reading frame, 1712bp. (WT) the MDV 814-infected CEF cells, the fragment of US2 region, 1180 bp; (NC) negative control.

Transfer plasmid pUAB-gpt-HA and MDV 814 DNA were used to co-transfect chicken embryo fibroblasts (CEFs) to obtain recombinant MDV-HA (rMDV-HA). At 5 d post co-transfection of pUAB-gpt-HA and MDV-814, the transfected CEFs exhibited the cytopathic effect (CPE). Highly purified rMDV-HA was obtained after seven rounds of selection. A ?4.6 kb PCR segment that covered the HA expression cassette and gpt selective marker was detected and an approximate 1.1 kb of US2 gene was undetectable in rMDV-HA DNA sample, but the 1.1 kb of US2 gene was detected in MDV 814 DNA sample. Furthermore, a 1.7 kb of HA gene was detected in rMDV-HA, but not in MDV 814 DNA sample (Fig. 1).

### Characterization of Recombinant rMDV-HA

**Figure 2:**
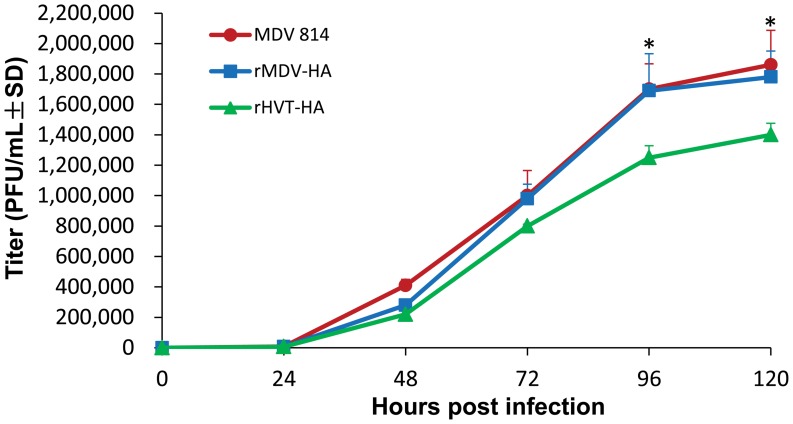
Growth curves (one-step growth kinetics) of rMDV-HA, MDV 814 and rHVT-HA. After inoculation of chickens with 100 PFU of each virus, virus titers were determined at different times and steadily increased from 48 to 96 h post-infection until maximal titers were reached. The data of the growth curve of rHVT-HA were cited from reference [18]. Stars indicate that the differences were significant between the groups (P<0.05).

After rMDV-HA was purified, the plaque size and growth curves of rMDV-HA (one-step growth kinetics) were determined and compared with those of MDV1 strain 814. The plaque sizes of rMDV-HA were similar to those of MDV1 strain 814 at 72 h post infection. Growth dynamics results indicated that the time course of rMDV-HA plaque development and plaque sizes were similar to those of wild type MDV1 strain 814 (Fig. 2).

The rMDV-HA virus titers steadily increased from 48 to 96 h post infection until titers peaked. At 96 and 120 hours post infection, the virus titers of rMDV-HA were significantly higher than those of rHVT-HA reported previously [30] (Fig. 2).

### Detection of Expressed Recombinant HA Protein

**Figure 3:**
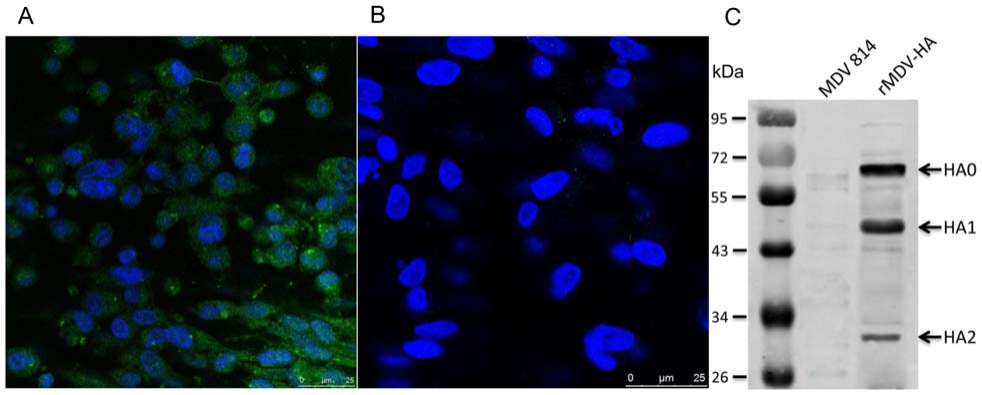
Immunofluorescence and Western blot analyses of recombinant HA protein expressed in rMDV-HA infected CEF cells. AIV-specific chicken serum antibodies bound to the rMDV-HA infected CEF cells (A) by indirect immunofluorescence, but not to the CEF cells infected with MDV 814 (B) 72 h post-infection**.** CEF cells infected with rMDV-HA and non-infected cells probed with an AIV-specific chicken serum and IRDye™ 800-labeled polyclonal rabbit anti-chicken IgG (1∶4000). HA-specific bands corresponding to the cleaved HA1 and HA2 were detected in preparations of rMDV- HA-infected cells by Western blot, but not in MDV 814 infected cells (C).

To confirm the expression of the recombinant HA protein, CEF cells infected with purified rMDV-HA on the 6-well plate were detected by indirect immunofluorescence test (IIFT). Green fluorescence was detected in rMDV-HA-infected cells with chicken anti-H5 AIV HA serum. CEFs infected with MDV1 strain 814 were negative by IIFT. Blue fluorescence was detected in nuclei of the cells after DAPI staining (Fig. 3).

Western blot analysis showed that three anti-HA immunoreactive bands, which corresponded to the molecular weight of the intact HA precursor HA0, the cleaved products HA1 and HA2, were observed in the infected cell extract sample, implying that recombinant HA protein was well expressed in CEFs infected with rMDV-HA. In contrast, no band was detected in cells infected with MDV strain 814 (Fig. 3).

### Viremia Levels of Chickens Infected with rMDV-HA

**Figure 4:**
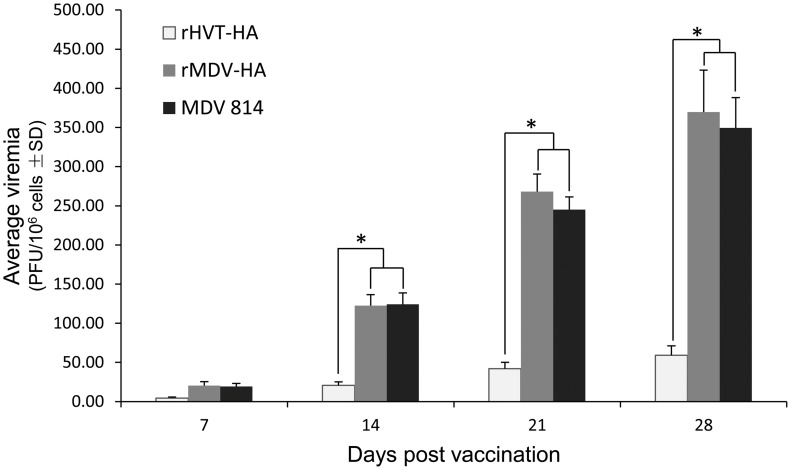
Comparison of viremia levels between rMDV-HA, MDV 814 and rHVT-HA. One-day old chicks were vaccinated with either rMDV-HA or MDV 814, and bled on 7, 14, 21 and 28 days post infection for determination of viremia. The data of the viremia level of rHVT-HA were cited from reference [18]. Stars indicate that the differences were significant between the groups (P<0.05).

To investigate the replication capability of rMDV-HA *in vivo*, the viremia levels in five chickens from each group were determined on 7, 14, 21 and 28 days post infection (Fig. 4). The viremia assay showed that there were no significant differences (P>0.05) in replication between MDV1 strain 814 and rMDV-HA during the whole experimental period. To compare the growth kinetics of rMDV-HA and rHVT-HA, the data on the viremia level of rHVT-HA were cited from our previous study [30] and analyzed, which showed that the viremia levels of rMDV-HA were significantly higher than those of rHVT-HA on days 14, 21 and 28 post infection.

### Protection of Chickens against Virulent MDV

**Figure 5:**
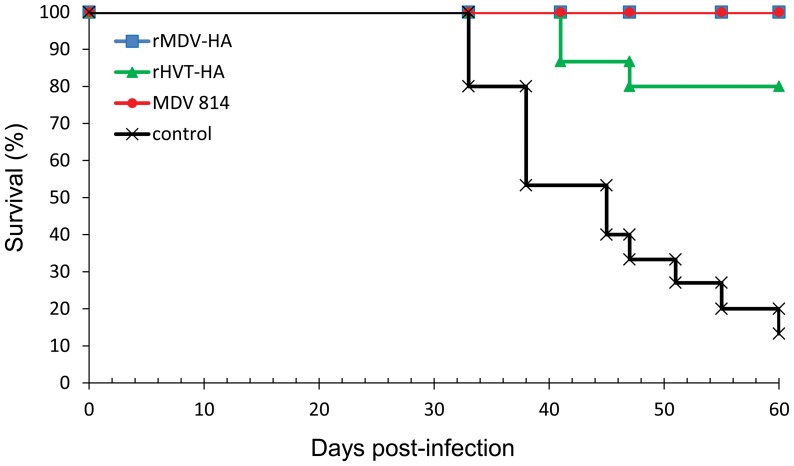
Protection of vaccinated chickens against virulent MDV challenge. Cumulative survival of chickens from unvaccinated chickens or the chickens vaccinated with rMDV-HA, rHVT-HA or MDV 814 during the 60 day evaluation period after chickens were challenged with virulent MDV strain J-1.

Vaccinated and control chickens were challenged with virulent MDV1 strain J-1 at 14 days post vaccination and data of the cumulative survival rates and gross/histological lesions were recorded. Four weeks after challenge, evidence of MD was observed in control chickens and all these control chickens developed MD and 86.7% of control chickens died during the 60-day period (Fig. 5). Post-mortem examination of these control chickens showed evidence of lymphoid tumors in several visceral organs. All the chickens vaccinated with the rMDV-HA or MDV1 strain 814 did not show any clinical signs and had no gross/histopathological tumors (a protective index of 100). In contrast, 26.7% (4/15) of rHVT-HA vaccinated chickens were detected with MD (a protective index of 73.3) and 20% (3/15) of vaccinated chickens died during the challenge experiment (Table 1).

**Table 1:** Protective efficacy of vaccines against virulent MDV challenge.

Vaccine	Number of chickens/group	MD mortality	MD%	Protection Index (PI) %
rHVT-HA	15	3	26.7	73.3
rMDV- HA	15	0	0	100
MDV 814	15	0	0	100
Control	15	13	100	0

MD (%) indicates the percentage of MDV-infected chickens that died after challenge with MDV strain J-1 or developed gross tumors prior to experimental termination.

### Vaccine Efficacy against Lethal H5N1 AIV Challenge

Before challenge, serum samples were collected weekly from vaccinated chickens and tested for H5N1 hemagglutination inhibition (HI) antibody titers to examine whether levels of HA-specific antibodies in rMDV-HA- or rHVT-HA-vaccinated chickens correlated with the protective efficacy of these vaccines against HPAIV. As shown in Table 2, HI antibody was undetectable in either rMDV-HA or rHVT-HA vaccinated chickens on 7 days post vaccination and increased gradually during the rest of experimental period. The mean HI antibody titers (log_2_) induced by rMDV-HA were 1.4±0.55 and 2.6±0.55 at 21 and 28 days post vaccination, respectively, which were significantly lower than the titers in rHVT-HA vaccinated group at corresponding days.

**Table 2:** Results of HI test of sera from chickens vaccinated with recombinant or MDV 814 vaccines.

Vaccine formulation tested	Log_2_ HI titer at different days post-vaccination(mean±SD)			
	7	14	21	28
rHVT-HA	0	1.6±0.90	3.2±0.84^A^	3.6±0.55^A^
rMDV-HA	0	0.8±0.84	1.4±0.55^B^	2.6±0.55^B^
MDV 814	ND	ND	ND	ND

ND = not determined. Different uppercase superscript letters indicate a significant difference (P<0.05) between groups on respective rows.

When the chickens were challenged, 12/15 (80%) of rMDV-HA vaccinated chickens were protected and virus was isolated from both tracheae and cloacae at 3 and 5 day post challenge whereas 10/15 (67%) of the chickens vaccinated with rHVT-HA were protected and viral shedding was detected till 7 day post challenge. All the chickens in the unvaccinated control group died within 48 h following the challenge (Table 3).

**Table 3:** Protective efficacy of vaccines against HPAIV H5 challenge in chickens.

Vaccine formulationtested	Virus isolated from collected swabs (shedding/total [log 10 EID_50_])^a^						
	Day 3 p.c.		Day 5 p.c.		Day 7 p.c.		Survival/total
	Oropharyngeal	Cloacal	Oropharyngeal	Cloacal	Oropharyngeal	Cloacal	
rHVT- HA	5/14(2.1±0.3)	2/14(2.4±0.2)	3/12(1.7±0.4)	1/12(2.3±0.1)	1/10(1.8±0.3)	None	10/15
rMDV-HA	4/15(2.4±0.3)	2/15(1.8±0.4)	1/13(2.7±0.3)	None	None	None	12/15
MDV 814	–b	–b	–b	–b	–b	–b	0/15

a Oropharyngeal and cloacal swabs were collected on days 3, 5, and 7 post challenge (p.c.) and titrated in SPF eggs.

b All chickens in this group died before day 3.

## Discussion

Many recombinant herpesvirus vectored vaccines have been developed and the licensed vaccine Vaxxitex^R^HVT+IBD has demonstrated the promising potential of herpesvirus vectors in the development and application of recombinant herpesvirus vaccines [31]. Since avirulent MDV-1 strains induce better protection against virulent MDV than HVT vaccine strain [13], [14] and there are few data on MDV-1 vector based AI vaccines, attenuated MDV-1 strain 814 expressing AIV H5HA (rMDV-HA) was developed in this study and preliminary experiments indicated US2 gene insertion did not affect the rMDV growth in CEFs as previously described for rHVT-HA [30]. Results from viral growth curve analyses in CEF and viremia demonstrated that growth kinetics and plaque sizes of rMDV-HA infected CEFs were similar to those of parental MDV-1. Western blot analysis indicated that immunogenic HA protein was successfully expressed in rMDV-HA-infected CEFs. In the virus challenge trial using HPAIV, 80% of rMDV-HA vaccinated chickens were protected whereas 67% of the chickens vaccinated with rHVT-HA were protected as reported previously [30] indicating that rMDV-HA induced better protection against AIV challenge than rHVT-HA.

There are several factors affecting efficacy of the recombinant MDV serotype 1 vaccines. The insertion site of the foreign gene affects the immunogenicity and vaccine efficacy of recombinant MDV. The unique short (US) 1, US2, US10 and thymidine kinase genes on the herpesvirus genome have been defined as ‘nonessential’ for viral replication in cell cultures [32], [33], [34]. The US2 gene on HVT [35] and MDV1 [19] and the US10 gene on HVT [22] and MDV1 [28] have been used as insertion sites for foreign genes in development of recombinant HVT or MDV, respectively. Our previous study demonstrated that US2 gene is a more suitable insertion site for foreign genes than US10 gene in the development of recombinant alphaherpesvirus vaccines [30]. In this study, rMDV-HA exhibited the same results with wtMDV in viral replication *in vitro* and protective efficacy, confirming our previous finding that US2 gene is a more ideal site for foreign gene insertion in alphaherpesviruses for development of effective recombinant vaccines.

For evaluation of protective efficacy of recombinant vaccine against MDV infection in this study, all chickens were challenged with the virulent MDV strain J-1. MDV strain J-1 is a reference virulent MDV strain isolated from Beijing district in China and is usually chosen as a challenge strain in MDV vaccine trials [15], [16]. In this study chickens immunized with rMDV-HA induced full protection against MDV strain J-1 challenge whereas partial protection (73.3%) was conveyed in chickens immunized with rHVT-HA, indicating that rMDV-HA vaccine induced better protection against MDV infection than rHVT-HA vaccine.

Chickens vaccinated with rMDV-HA vaccine in this study induced 80% protection against HPAIV compared to 67% protection induced by rHVT-HA. These results demonstrated that rMDV-HA induced more effective protection against HPAIV challenge than rHVT-HA. When compared to inactivated avian influenza virus vaccines, the rMDV-HA vaccine has the advantages to allow serological discrimination between vaccinated and field-virus infected animals by the absence or presence of antibodies against NA and NP, which are detectable by standard diagnostic tests.

Some avian viruses have been used as viral vectors to develop recombinant AI vaccines. Recombinant FPV [36] or NDV [20], [37] expressing AIV HA gene were generated and induced significant levels of protection against HPAIV challenge. La Sota NDV vaccine expressing AI H5 (rLH5-5) was licensed and commercialized in China in 2005 [38], [39]. Another avian herpesvirus, infectious laryngotracheitis virus, was also used for developing recombinant vaccines expressing HA genes encoding H5 [40] and H7 [41] of HPAIV, and the immunized animals produced specific antibodies against ILTV and AIV HA and were protected against challenge infections with either virulent ILTV, or two different highly pathogenic AIV strains. Recombinant duck enteritis virus vectored live vaccine provided fast and complete protection against lethal H5N1 avian influenza virus challenge in ducks [42]. Chickens vaccinated with rHVT bivalent vaccine expressing AI H5HA [30] or H7HA [18] were protected against AI and MD infection. In this study, rMDV-HA induced full protection against virulent MDV challenge and more efficient protection against AI infection than rHVT-HA that was developed previously [30]. According to data from viral growth kinetics assay *in vitro* and viral viremia level assay, the virus load of rMDV-HA was significantly higher than that of rHVT-HA in both assays, which might account for the better protection against virulent MDV and HPAIV challenges in rMDV-HA group. Previous work has indicated that limited virulence might enhance the efficacy of MDV1 vaccines, perhaps through greater invasiveness, which may be associated with higher viremia titers [43], [44]. However, the mean HI antibody titers induced by rMDV-HA were significantly lower than those in rHVT-HA vaccinated group, but rMDV-HA induced better protection than rHVT-HA, indicating that there is no direct correlation between HI antibody induced by herpesvirus based AI vaccine and the protection rate. Cellular immune response possibly plays an important role in protection induced by herpesvirus based vaccines. Additional studies are currently underway to evaluate the role of cellular immune response in this study.

In summary, we successfully constructed the first recombinant MDV serotype 1 vaccine expressing AIV-HA that provided good protection against challenges with virulent MDV and HPAI H5 viruses. Thus, an attenuated MDV1 recombinant vaccine expressing HA may be used as a recombinant live virus vaccine against AI and MD. We also demonstrated MDV-1 814 vector vaccine expressing AIV H5HA induced better protection than HVT-based vector vaccine by comparing protective efficacy of rMDV-HA and rHVT-HA. These results will provide important information for development of recombinant herpesvirus vaccines expressing the protective genes of economically important viruses in poultry industry.

## Materials and Methods

### Ethics Statement

Animal experiments were approved by Animal Ethics Committee of Harbin Veterinary Research Institute of the Chinese Academy of Agricultural Sciences (CAAS) and performed in accordance with animal ethics guidelines and approved protocols. The Animal Ethics Committee approval number was Heilongjiang-SYXK 2006-032.

### Viruses and Cells

The MDV vaccine strain 814 is a vaccine strain developed and widely used in China [45] and the twentieth chicken embryo fibroblast (CEF) passage stock was used in our study for the construction of the recombinant viral vector. The virulent MDV J-1 strain is a virulent reference strain in China [46] and the tenth duck embryo fibroblast passage stock was used as the challenge virus in protection studies. Both viral isolates were obtained from the Avian Infectious Diseases Laboratory of Harbin Veterinary Research Institute of CAAS and were propagated in CEFs prepared from 10-day-old specific-pathogen-free (SPF) embryos provided by State Key Laboratory of Veterinary Biotechnology, Harbin Veterinary Research Institute of CAAS. rHVT-HA (rHVT-US2-HA) was constructed in our laboratory in previous study [30]. The influenza virus HPAIV A/Goose/HLJ/QFY/2003 (H5N1) [47] and HA gene of A/goose/Guangdong/3/96 (H5N1) [48] isolated in China were kindly provided by National Avian Influenza Reference Laboratory of China and propagated in the allantoic cavities of 10-day-old SPF chicken embryonated eggs for challenge in animal studies.

### Construction of the Transfer Vector pUAB-gpt-HA

The plasmid pUAB-gpt was constructed as described previously [49], which contained 2.1 and 3.0 kilo base pair (kb) fragments flanking the MDV 814 US2 gene and the *E. coli* derived selective gpt marker under the control of the hCMV immediate-early promoter. The plasmid pN1-HA containing the ORF of the HA gene of A/goose/Guangdong/3/96 (H5N1) was constructed in our laboratory previously [30]. The HA cassette was amplified using primers (forward: 5′-CGGCGGTTAATTAACGCCATGCATTAGTTATT-3′and reverse: 5′-CGGCGGTTAATTAACGCTTACAATTTACGCCT-3′) and inserted into *Pac*? site of plasmid pUAB-gpt to obtain the transfer plasmid pUAB-gpt-HA.

### Co-transfection and Generation of rMDV-HA

The recombinant MDV was generated as described previously [49]. Briefly, primary CEFs were co-transfected with 1 µg of pUAB-gpt-HA and 5 µg of MDV 814 DNA using Lipofectamine™ 2000 (Invitrogen, Beijing, China) according to the manufacturer’s instructions. When plaques were observed, virus-containing cells were passaged repeatedly in selection medium using Eco-gpt selectable marker. For the enrichment and purification of rMDV-HA, the transfected cells with visible CPE were picked and selected for the survival of rMDV-HA in the selection medium. Briefly, the selection medium contained mycophenolic acid (350 µg/ml), xanthine (70 µg/ml) and hypoxanthine (100 µg/ml) (Sigma, Shanghai, China). The single plaque was picked out, harvested with trypsin and serially subcultured on secondary CEFs in the selection medium until no cells with CPE were observed in selection medium suspensions. As part of the selection process, total DNA extracted from infected cells was detected by PCR with primers based on the US2 gene or HA gene. PCR amplifications that covered the US2 region (forward: 5′-AAAAAGATTATTGGTGGAGGTGAAG-3′and reverse: 5′- GTAGCAAG TAGGTCTGTCGAATAACAG-3′) or HA ORF (forward: 5′-ATGGAGAGAATA GTGCTTCTCC-3′and reverse: 5′-CAAATTCTGCATTGTAACGAT -3′) were performed on DNA sample. All the PCR products were verified by sequencing.

### Plaque Assays and One-step Growth Kinetics

The growth dynamics and plaque sizes of rMDV-HA were compared with those of wild type MDV 814. One-step growth analyses of rMDV-HA were performed as described previously [49], [50]. Briefly, 100 plaque-forming unit (PFU) of each of rMDV-HA and wtMDV were simultaneously used to infect different groups of fresh CEF cells. At 0, 24, 48, 72, 96 and 120 h post-infection, virus-infected CEFs were harvested and serial 10-fold dilutions were added in triplicate onto the forty-eight-well plates of CEFs. The titers of the virus at each time point were calculated from the number of PFU from each of the dilutions and the growth curves of rMDV-HA and wtMDV were determined.

### Indirect Immunofluorescence Tests and Western Blot Analyses

For detection of rMDV-HA HA gene expression by indirect immunofluorescence tests *in vitro*, CEFs infected with rMDV-HA were fixed with ice-cold ethanol after CPE was observed. The wells were overlaid with polyclonal chicken antibodies produced by vaccination with A/goose/Guangdong/3/96 (1∶100) and incubated at 37°C for 1 h. The cells were washed three times and subsequently incubated at 37°C for 1 h with anti-chicken IgY (IgG) (whole molecule)-FITC antibody produced in rabbit (1∶300) (Sigma, Shanghai, China). The cells were then incubated with DAPI staining solution [51]. wtMDV-infected CEF cells and non-infected CEF cells were also stained. The sections were imaged in a Leica TCS SP5 confocal microscope equipped with 488- and 340–365-nm laser excitation (Leica Microsystems, Mannheim, Germany).

CEFs infected with rMDV-HA or wtMDV were harvested 3 days after infection. Lysates of cells were separated on discontinuous 10% SDS polyacrylamide gels and electrotransferred to nitrocellulose membranes, followed by 0.1% trypsin for 30 min. The blots were incubated for 1 h with chicken anti-H5N1 AIV serum (1∶300), and then with IRDye™ 800-labeled polyclonal rabbit anti-chicken IgG (1∶4000) (Li-Cor, Lincoln, NE). Antibody binding was detected by Odyssey® Infrared Imaging System (Li-Cor, Lincoln, NE).

### Determination of Viremia

The level of rMDV-HA viremia was determined as previously described [30]. Briefly, 40 chickens were divided into two groups and vaccinated intramuscularly with a total of 3000 PFU of either rMDV-HA or MDV 814. Blood samples in anticoagulants were collected from five chickens of each group weekly and 5 ml of blood from each chicken was mixed with 5 ml RPMI 1640 medium and 3 ml Histopaque 1077 (Sigma, Shanghai, China). The leukocytes were recovered by centrifugation and counted. A total of 2×10^6^ leukocytes were cultivated in duplicate onto 60-mm plates with CEF monolayers. Dishes were stained with crystal violet and plaques were counted following CPE appearance.

### Protection against Virulent MDV

Forty-five 1-day-old SPF chicks were randomly divided into three groups (n = 15) and vaccinated intramuscularly with rMDV-HA, rHVT-HA or MDV 814 (3000 PFU/chick). Fifteen negative control chicks were inoculated with non-infected CEFs. At 14 d post vaccination, chickens were challenged by intra-abdominal injection of 1000 PFU of virulent MDV J-1 virus. The chickens were observed daily for clinical symptoms, and monitored for mortality for 60 days after the challenge. Both dead chickens and surviving chickens that were euthanized were examined for gross and histopathological lesions. The percentage of gross MD was calculated for each test group as the number of chickens with gross MD lesions divided by the number at risk (survivors plus MD deaths)×100. Vaccinal immunity to MD was expressed as a protective index calculated as the percentage of gross MD in non-vaccinated challenged control chickens minus the percentage of gross MD in vaccinated, challenged chickens divided by the percentage of gross MD in nonvaccinated challenged control chickens and multiplied by 100 [52].

### Protection against HPAIV

A total of 45 day-old SPF chicks were used in this experiment. Each of 15 chicks was vaccinated intramuscularly with a total of 3000 PFU of either rMDV-HA, rHVT-HA or MDV 814. Before challenge, sera were collected weekly and tested by H5N1 HI antibody assays, respectively. Four weeks post vaccination, all chickens were challenged intranasally with 10^5^ ELD_50_ of HPAIV H5N1 A/Goose/HLJ/QFY/03 viruses. The chickens were observed daily for clinical symptoms, and oropharyngeal and cloacal swabs of the chickens were taken on days 3, 5, and 7 post challenges for titration of shed AIV. Chickens were monitored for mortality for 2 weeks after challenge. After 8 weeks all surviving animals were euthanized and examined for pathological alterations.
